# Impact of Omicron variant infection on the liver, kidney, and coagulation system in patients undergoing elective surgery: a retrospective case-control study

**DOI:** 10.7150/ijms.88727

**Published:** 2024-02-17

**Authors:** Xiaojuan Xiong, Rui Li, Haoyu Pei, Qingxiang Mao

**Affiliations:** Department of Anesthesiology, Army Medical Center of PLA, Daping Hospital, Army Medical University, 10 ChangjiangZhilu, Yuzhong District, Chongqing 400042, China.

**Keywords:** coagulation parameters, complete blood count, elective surgery, liver function, kidney function, Omicron variant

## Abstract

**Purpose:** We aimed to investigate the impact of Omicron variant infection on the perioperative organ function in patients undergoing elective surgery.

**Methods:** A total of 5029 patients who underwent elective surgery between October 2022 and January 2023 at our hospital were enrolled. Among them, the patients who underwent elective surgery between October 2022 and November 2022 composed Group 1 (not infected with the Omicron variant) the control group; those who underwent elective surgery between December 2022 and January 2023 composed Group 2 (one month after Omicron variant infection) the experimental group. We further divided the patients into two subgroups for analysis: the tumor subgroup and the nontumor subgroup. Data on organ system function indicators, including coagulation parameters, liver function, complete blood count (CBC), and kidney function, were collected before and after surgery. Differences between the two groups were subsequently analyzed via binary logistic regression analysis.

**Results:** Compared with those in the uninfected patient group, the following changes were observed in patients with Omicron variant infection who underwent elective surgery one month after infection: prothrombin activity (PTa), prothrombin time (PT), fibrinogen, albumin/globulin, alanine aminotransferase (ALT), mean corpuscular hemoglobin concentration (MCHC), platelet (PLT), and anemia were increased AST/ALT, indirect bilirubin (IBILI), eosinophils, and uric acid were decreased before surgery; and lung infection/pneumonia and fibrinogen were increased, while AST/ALT, globulin, total bilirubin (TBIL), white blood cell count (WBC), and uric acid were decreased after surgery. There was no significant difference in the mortality rate or length of hospital stay (LOS) between the two groups. Subgroup analysis revealed elevated monocyte, PLT, and fibrinogen classification, levels and decreased globulin, prealbumin (PBA), eosinophil, and uric acid levels in the tumor subgroup of patients who underwent elective surgery one month after Omicron infection compared with those in the uninfected patients. Compared with the nontumor subgroup, fibrinogen levels, lung infection/pneumonia, TBIL, and PLT count were increased in the uninfected patients, while the globulin and eosinophil levels were decreased.

**Conclusion:** Compared with uninfected patients, patients who underwent elective surgery one month after Omicron variant infection exhibited minimal changes in perioperative coagulation parameters, liver function, CBC counts, and kidney function. Additionally, no significant differences in postoperative mortality or LOS were observed between the two groups.

## Introduction

SARS-CoV-2, a virus with a single-stranded RNA genome and a distinctive spike protein on its surface, causes COVID-19, which affects not only the respiratory system but also other major organs, such as the heart, brain and liver 1,2. On November 24, 2021, the South African Minister of Health reported the emergence of a new and rapidly spreading variant of SARS-CoV-2 discovered by the Network for Genomic Surveillance in South Africa 3. Two days later, the WHO officially disclosed the emergence of this novel SARS-CoV-2 variant of concern and named it Omicron 4. As of May 21, 2023, more than 766 million confirmed cases and more than 6.9 million deaths have been reported globally 5. On January 8, 2022, the first case of indigenous Omicron variant infection was confirmed in Tianjin. The variant subsequently spread across China 6. In December 2022, as China adjusted its COVID-19 policies and significantly eased controls and restrictions, a large population was infected with the Omicron variant in a very short period 7. These patients in Chongqing were predominantly infected with the Omicron-BA.5.2 variant, with some infections involving Omicron-BF7 8.

Scientists in South Africa have shown that the Omicron variant has decreased virulence, leading to fewer severe cases and lower mortality rates when it became prevalent 9. Ferrandis et al. reported that hypercoagulability following infection could persist for one to three months, possibly up to six months 10. Wolff et al. reported that COVID-19 alters the levels of several laboratory parameters, mainly hepatic and kidney function markers. For example, markers of liver function, such as alanine aminotransferase (ALT), aspartate aminotransferase (AST), lactic acid, procalcitonin, and total direct and indirect bilirubin levels, may increase, whereas albumin levels decrease. Regarding kidney function, increased blood urea nitrogen and creatinine levels, proteinuria, and hematuria may occur 11. Zhang et al. reported that Omicron variant infection resulted in a substantial proportion of patients exhibiting signs of leukocytosis, neutrophilia, lymphocytopenia, monocytosis, and coagulopathy, while the levels and major functional indices of red blood cells (RBCs) and platelets (PLTs) were minimally reduced 12.

Limited research has been conducted to investigate the potential impact of Omicron variant infection on liver, kidney, and coagulation parameters before surgery, as well as its potential in exacerbation of worsening postoperative liver and kidney function in patients who underwent elective surgery one month after Omicron variant infection. We hypothesized that clinical indicators, including coagulation parameters, liver function, complete blood count (CBC), and kidney function, did not change significantly during the perioperative period in patients who underwent elective surgery one month after Omicron variant infection.

## Materials and methods

### Inclusion and exclusion criteria

The inclusion criterion was patients who underwent elective surgery at our hospital between October 2022 and January 2023.

The exclusion criteria for patients who were as follows: 1. were infected with the Omicron variant between October 2022 and November 2022; 2. had no infection with the Omicron variant between December 2022 and January 2023; 3. lacked laboratory test records before surgery; 3. had undergone emergency surgery; 4. were younger than 18 years of age. 5. presence of acute upper respiratory infection with typical catarrh symptoms (cough, rhinorrhea, sneezing, and nose congestion); 6. detection of clear rales on auscultation of the lungs; 7. fever exceeding 38 degrees Celsius; 8. oxygen saturation less than 92% during inhalation of air or an oxygenation index less than 300 on a blood gas test; and 9. a CT scan revealing manifestations of pneumonia.

### Research methods

Among 5732 patients who underwent elective surgery between October 2022 and January 2023, 5029 patients were enrolled. Based on their surgical data, the enrolled patients were divided into two groups: Group 1 (the control group) consisted of patients who underwent elective surgery between October 2022 and November 2022 and were not infected with the Omicron variant; Group 2 (the experimental group) included patients who underwent elective surgery between December 2022 and January 2023, one month after they contracted the Omicron variant. We further divided the patients into two subgroups for analysis: the tumor subgroup and the nontumor subgroup. Pre- and postoperative values of various indicators, including coagulation parameters, liver function, CBC, and kidney function, were collected and compared to determine the differences between the two groups. When the sample data loss exceeded 20%, the data were promptly discarded. When the sample size was less than 20%, SPSS software was used to impute the missing data. Given that this was a retrospective study, the Ethics Committee waived the requirement for obtaining informed consent forms from the patients. This study was approved by the Army Medical Center of PLA (ratification number is 2023 177) on May 4, 2023. The study was registered with the WHO International Clinical Trial Registration (ChiCTR2300071913).

During the pandemic, COVID-19 genome sequencing in Chongqing city was conducted by government-designated laboratories. All nasopharyngeal swab specimens from the patients in our hospital were randomly sampled and sent to the designed laboratory for whole-genome sequencing of the coronavirus. Therefore, according to the public information disclosed by the Chongqing City government, the patients included in this retrospective survey were predominantly infected with the Omicron-BA.5.2 variant, with some cases involving Omicron-BF7 8.

### Data collection

The electronic medical records and the surgical anesthesia system were used to query the clinical data of the patients. We collected basic demographic information about the patients, namely, age, sex, medical records, hypertension, diabetes status, coronary heart disease (CHD), chronic obstructive pulmonary disease (COPD), malignant tumor, renal insufficiency, lung infection/pneumonia, pleural effusion, length of stay (LOS), and mortality. The laboratory markers included coagulation parameters, liver function, CBC results, and kidney function (**Table 1**). Other medical indicators included D-D grade (>400 µg/L), activated partial thromboplastin time (APTT) classification (>40 s), thrombin time (TT) classification (>18 s), prothrombin activity (PTa) classification (>120%), PT classification (>13.8 s), and fibrinogen classification (>4 g/L). Liver injury was defined as follows: among these liver function test parameters, ALT, AST, TBA, alkaline phosphatase (ALP), gamma-glutamyl transferase (GGT), or total bilirubin (TBIL) exceeded the upper limit of normal value or albumin was lower than the lower limit of normal value 13. The patients were classified according to this definition as ALT, AST, ALP, GGT, or TBIL. According to the 2011 WHO standard (and the reference index of our laboratory), anemia was defined as hemoglobin level <130 g/L males and <120 g/L in females 14. The results of perioperative lower extremity ultrasounds were collected.

### Statistical analyses

All the statistical analyses were performed using SPSS 26.0 software (IBM Corp., Armonk, NY, USA). Chi-square tests or Fisher exact probability tests were used to process the data, and the results are expressed as percentages (%). The Shapiro‒Wilk test was used to determine whether the measurement data were normally distributed. The normality of the distribution of the measured data was tested by an independent sample t test, and the results are expressed as the mean (±) standard deviation (x ± s). The Mann‒Whitney U test was used to test the nonnormal distribution of the measurement data, and the results are expressed as the median (quaternary) [M (Q1, Q3)]. In logistic regression, the significant values of the univariate logistic regression were tested for collinearity, and the indicators with a variance inflation factor (VIF) greater than ten were eliminated before being included in the multivariate logistic regression. *P* < 0.05 was considered to indicate statistical significance.

## Results

### Basic information about the patients

A total of 5732 patients underwent elective surgery at our hospital between October 2022 and January 2023. Among them, 703 patients were excluded: 180 for lack of preoperative laboratory data, 17 who were under 18 years of age, 16 who were infected with the Omicron variant between October 2022 and November 2022, and 190 who were not infected with the Omicron variant between December 2022 and January 2023. The median period from Omicron variant infection to surgery was 30 days. The vaccination rate among all patients was up to 90.1% 8. Finally, 5029 patients were enrolled, representing a sample loss rate of less than 15%. Of the enrolled patients, 2071 were male, and 2958 were female (**Table 1**). The most common preoperative comorbidities were hypertension (724 patients, 14.4%), diabetes (521 patients, 10.18%), lung infection/pneumonia (199 patients, 3.96%), and CHD (136 patients, 2.7%) (**Table 2**) (**Table 3**).

The average LOS was 8.60 days for group 1, 8.66 days for Group 2 (*P*=0.839). In Group 1, the perioperative mortality rate was 0.13% (3093 out of 4), whereas in Group 2, the mortality rate was 0.36% (1931 out of 7) (**Table 3**). The chi-square test yielded a value of 2.97 (*P*=0.085). Univariate logistic regression analysis revealed the following: OR=2.81, 95% CI:0.822-9.623, *P*=0.099. Notably, there was no significant difference in the mortality rate or LOS between the two groups.

### Results of the univariate logistic regression analysis

**Figure 1:**
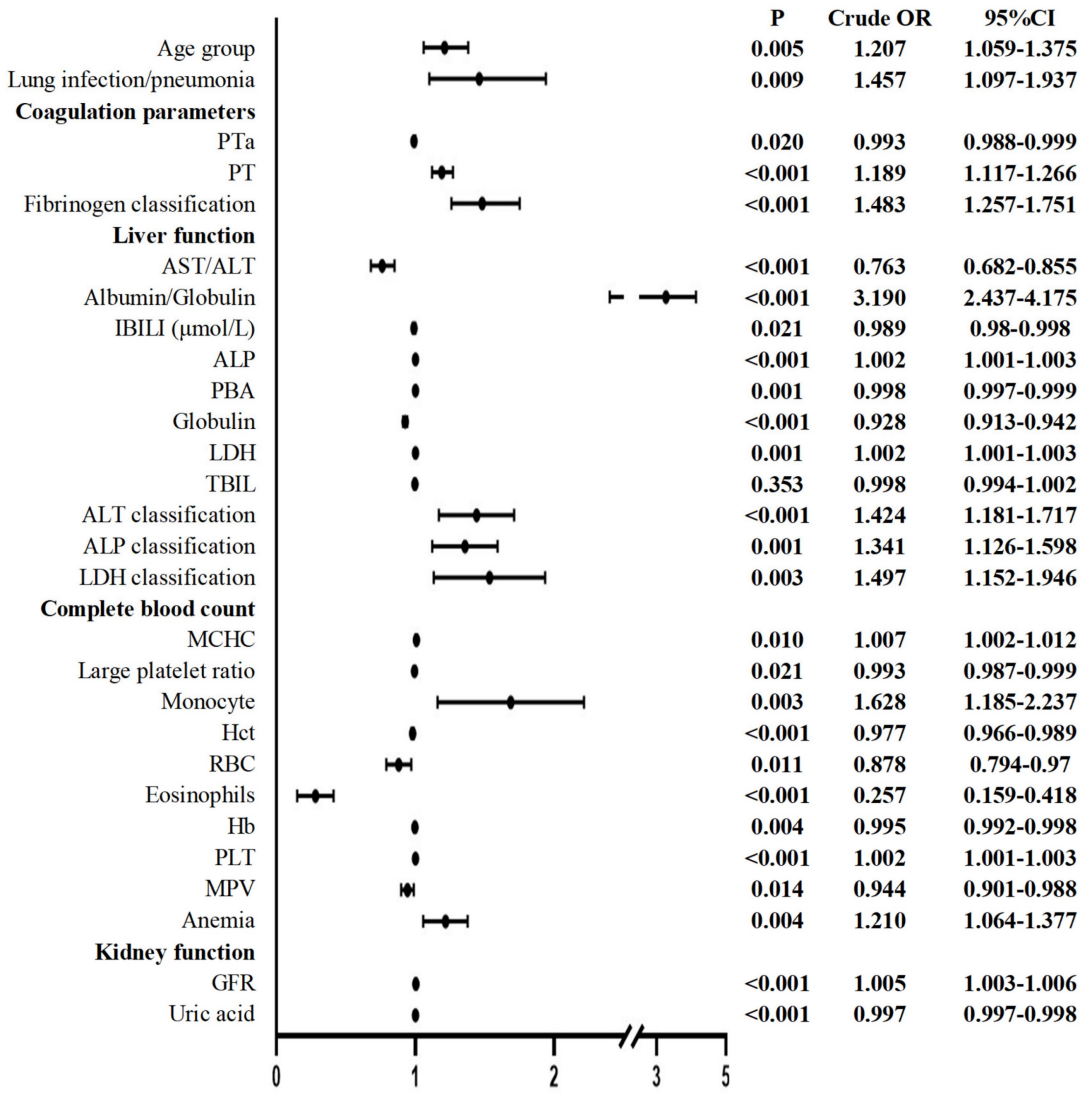
Preoperative univariate logistic regression of patients undergoing elective surgery in one month after infection with the Omicron variant and uninfected patients. ALP: Alkaline phosphatase; ALT: Alanine aminotransferase; AST: Aspartate aminotransferase; GFR: glomerular filtration rate; Hb: hemoglobin; HCT: Hematocrit; IBILI: Indirect bilirubin; MCHC: mean corpuscular hemoglobin concentration; MPV: mean platelet volume; PLT: Platelet; PT: Thrombin time; PTa: Prothrombin activity; RBC: Red blood cell count; TBIL: Total bilirubin; UA: Uric acid; PBA: Prealbumin; LDH: Lactate dehydrogenase. *P* < 0.05 was statistically significant.

The univariate logistic regression analysis of the preoperative values revealed that the following indices were significantly different (*P*<0.05): age group; lung infection**/**pneumonia; coagulation parameters: PTa, PT, fibrinogen; liver function: AST/ALT, albumin/globulin, IBILI, ALP, prealbumin (PBA), globulin, lactate dehydrogenase (LDH), ALT classification, ALP classification, LDH classification; CBC: mean corpuscular hemoglobin concentration (MCHC), large platelet ratio, monocyte, hematocrit (HCT), RBC, eosinophil, hemoglobin (Hb), and mean platelet volume (MPV); and kidney function: glomerular filtration rate (GFR) and uric acid (**Figure 1**). A multicollinearity analysis was then conducted for these indices. As a result, indices with a variance inflation factor (VIF) higher than 10, namely the platelet ratio, Hb concentration, and MPV, were excluded from subsequent multivariate logistic regression analysis.

**Figure 2:**
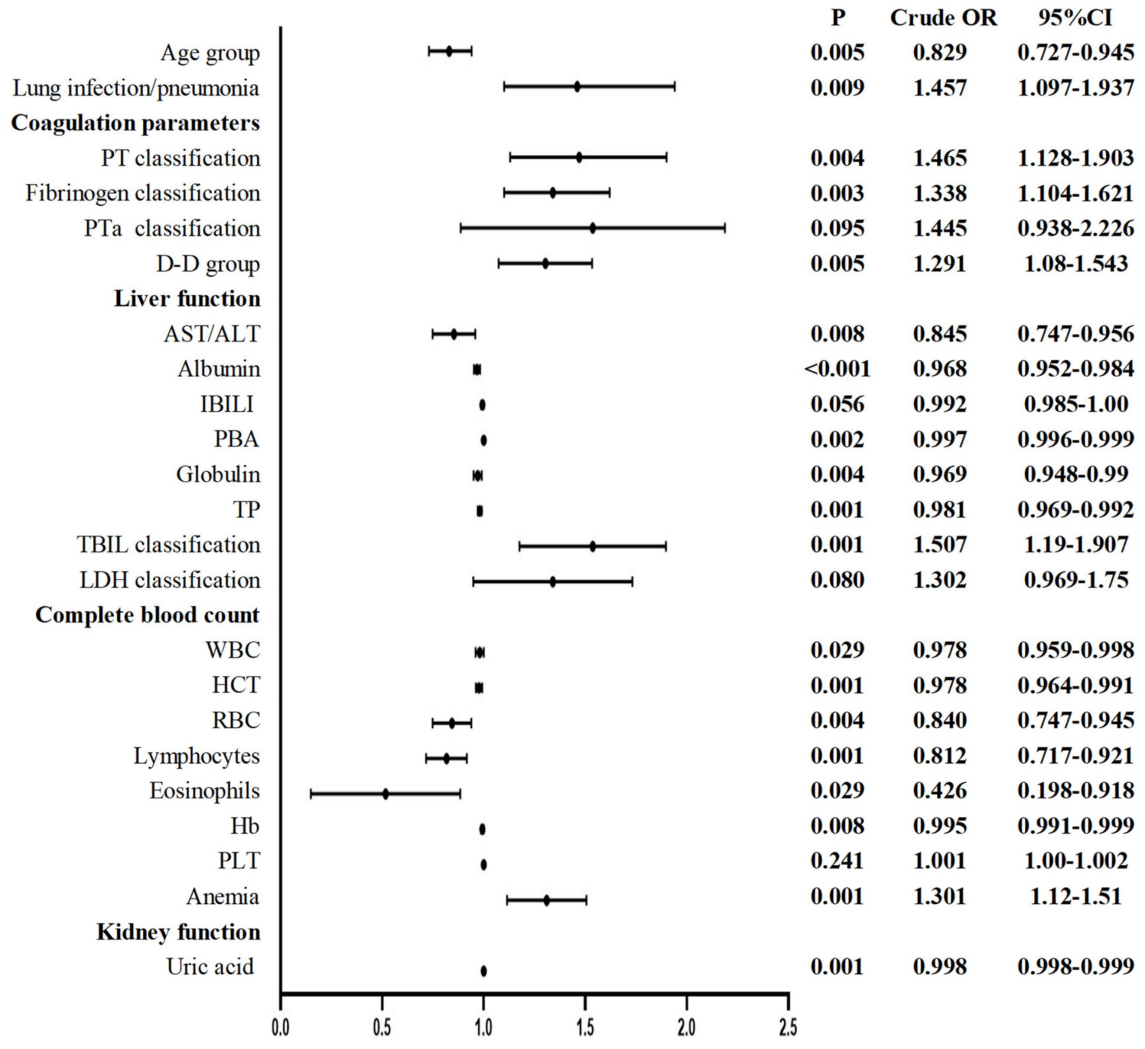
Postoperative univariate logistic regression of patients undergoing elective surgery in one month after infection with the Omicron variant and uninfected patients. AST: Aspartate aminotransferase; ALT: Alanine aminotransferase; HCT: Hematocrit; Hb: hemoglobin; IBILI: Indirect bilirubin; PLT: Platelet; PT: Thrombin time; PTa: Prothrombin activity; RBC: Red blood cell count; TBIL: Total bilirubin; WBC: White blood cell count; PBA: Prealbumin; LDH: Lactate dehydrogenase; TP: Total protein; RDW: Red cell distribution width. *P* < 0.05 was statistically significant.

The univariate logistic regression analysis of the postoperative values revealed that the following indices were significantly different (*P*<0.05): age group; lung infection/pneumonia; coagulation parameters: PT classification, fibrinogen classification, and D-D group; liver function: AST/ALT, albumin, PBA, globulin, total protein (TP), and TBIL classification; CBC: WBC, HCT, RBC, lymphocytes, eosinophils, Hb, and anemia; and kidney function: uric acid (**Figure 2**). An analysis of multicollinearity was conducted for these indices. Multicollinearity was subsequently assessed among these indices. As a result, indices with a VIF higher than 10, namely, TP, HCT, and Hb, were excluded from the subsequent multivariate logistic regression analysis.

### Results of the multivariate logistic regression analysis

**Figure 3:**
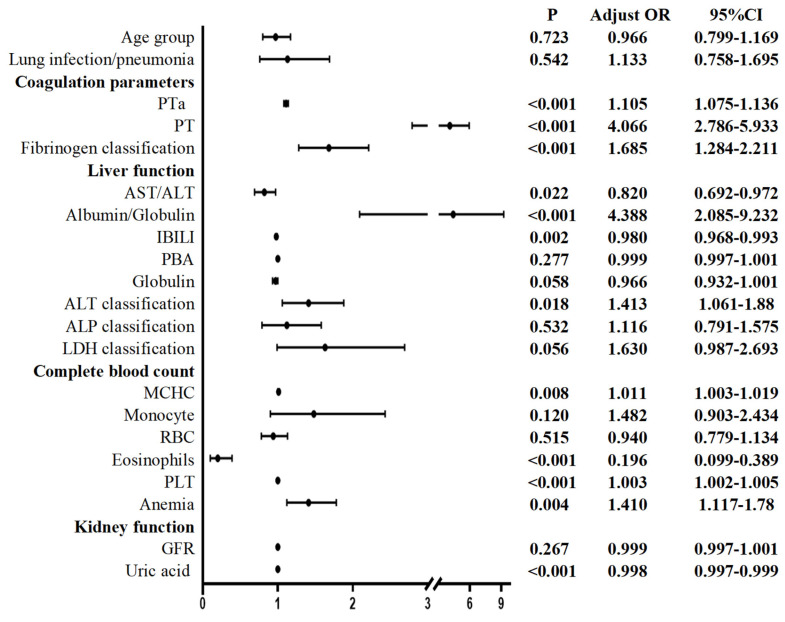
Preoperative multivariate logistic regression of patients undergoing elective surgery in one month after infection with the Omicron variant and uninfected patients. ALP: Alkaline phosphatase; ALT: Alanine aminotransferase; AST: Aspartate aminotransferase; GFR: glomerular filtration rate; IBILI: Indirect bilirubin; MCHC: mean corpuscular hemoglobin concentration; PLT: Platelet; PT: Thrombin time; PTa: Prothrombin activity; RBC: Red blood cell count; UA: Uric acid; PBA: Prealbumin; LDH: Lactate dehydrogenase. *P* < 0.05 was statistically significant.

Multivariate logistic regression analysis of the preoperative values revealed the following for Group 2 compared to the patients not infected with the Omicron variant: coagulation parameters (PTa, PT, fibrinogen classification), liver function (AST/ALT, albumin/globulin, IBILI, ALT classification), CBC (MCHC, eosinophils, PLT, anemia), and kidney function (uric acid) were considered to be significantly different (**Figure 3**).

**Figure 4:**
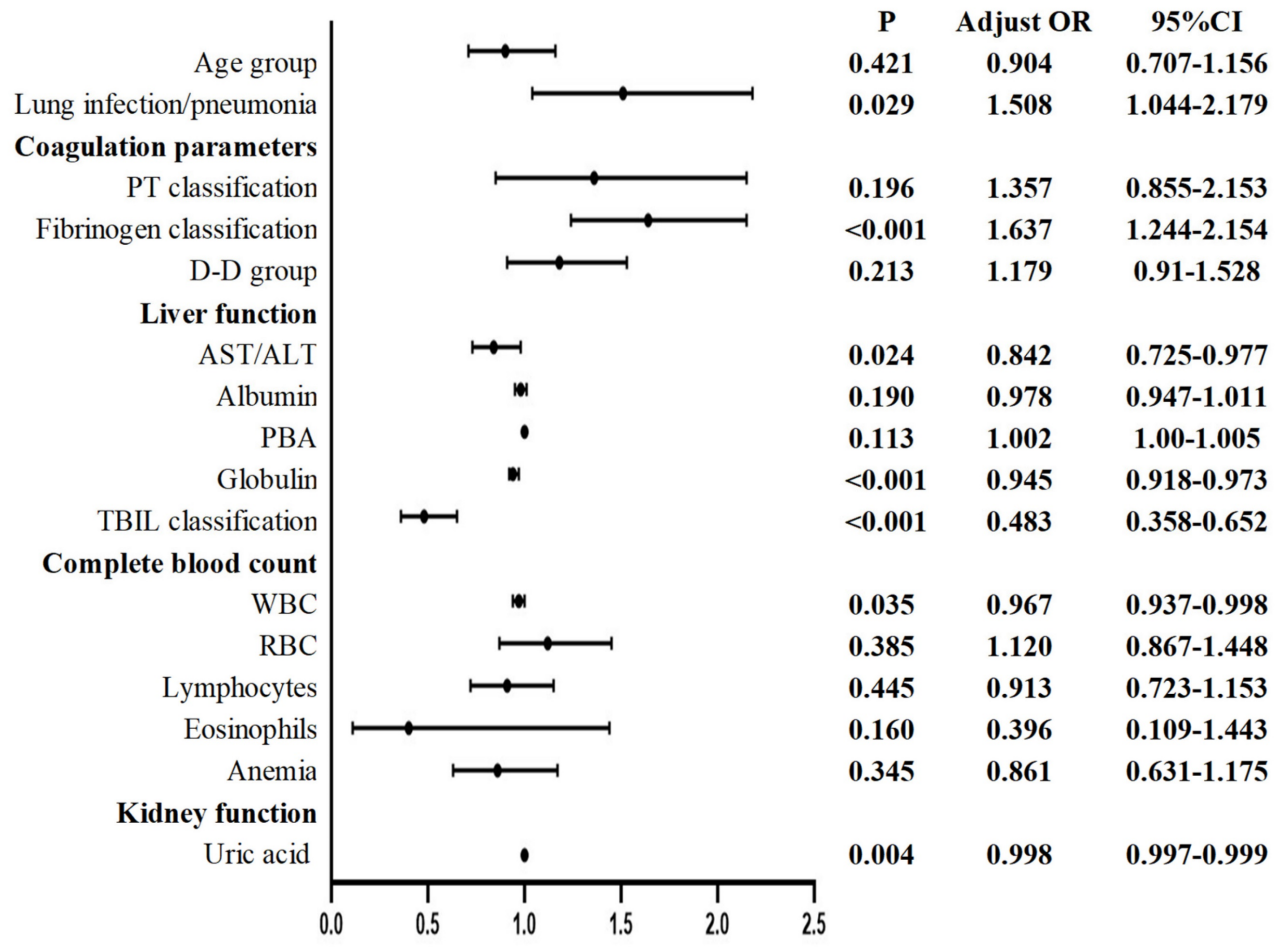
Postoperative multivariate logistic regression of patients undergoing elective surgery in one month after infection with the Omicron variant and uninfected patients. AST: Aspartate aminotransferase; ALT: Alanine aminotransferase; RBC: Red blood cell count; TBIL: Total bilirubin; WBC: White blood cell count; PBA: Prealbumin, PT: Thrombin time. *P* < 0.05 was statistically significant.

Multivariate logistic regression analysis of the postoperative values revealed the following for Group 2 compared with patients not infected with the Omicron variant: lung infection/pneumonia, coagulation parameters (fibrinogen classification), liver function (AST/ALT, globulin, TBIL classification), CBC (WBC), and kidney function (uric acid) were considered to be significantly different (**Figure 4**).

Compared with those in uninfected patients, the following changes were observed in the Omicron-infected patients one month after infection: before surgery, elevated PTa, PT, fibrinogen, albumin/globulin, ALT, MCHC, and PLT and anemia were observed; conversely, decreased AST/ALT ratio; IBILI, eosinophils, and uric acid were observed. After surgery, lung infection/pneumonia and fibrinogen were increased, while AST/ALT, globulin, TBIL, WBC, and uric acid were decreased.

### Subgroup analysis

**Figure 5:**
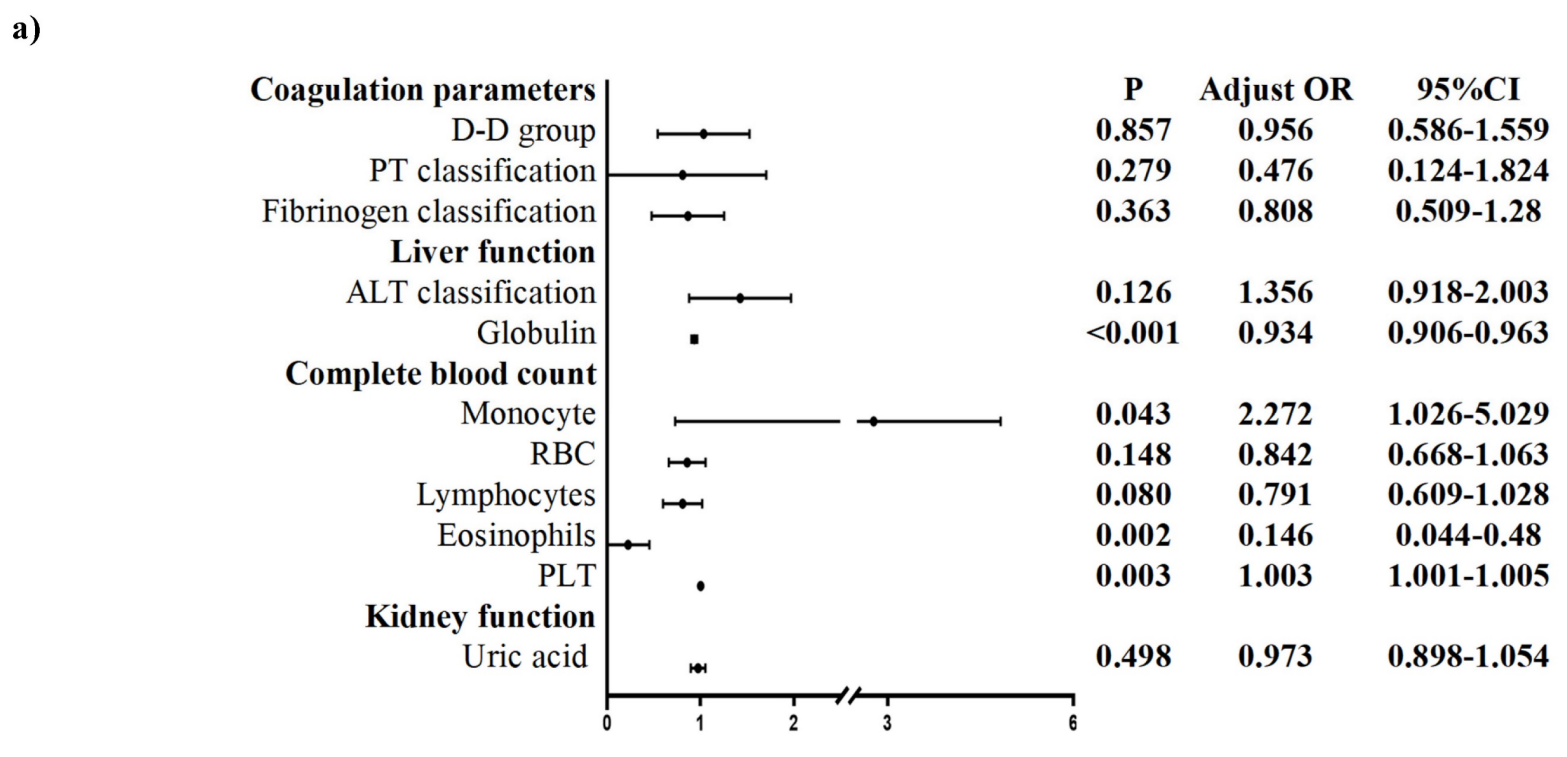

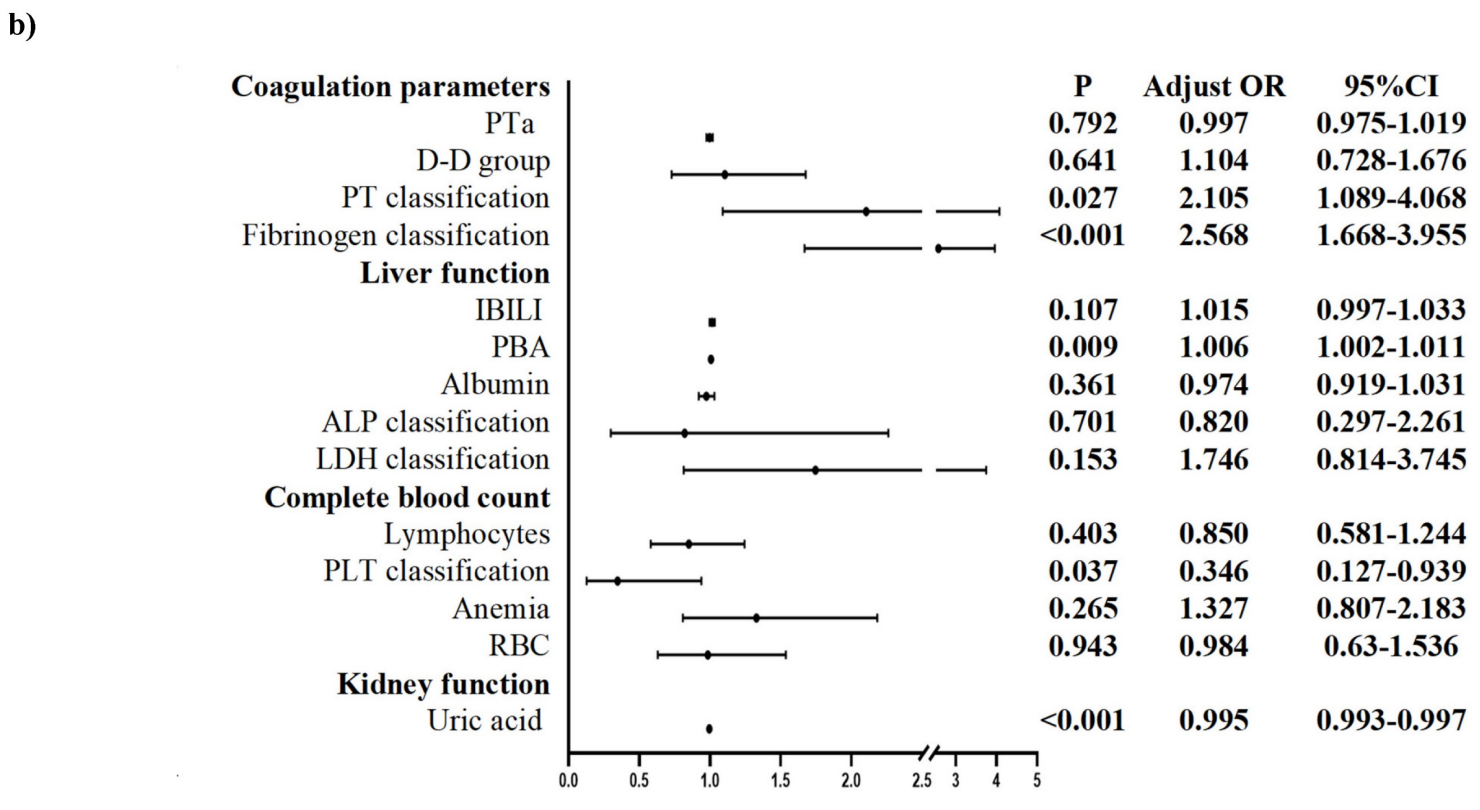

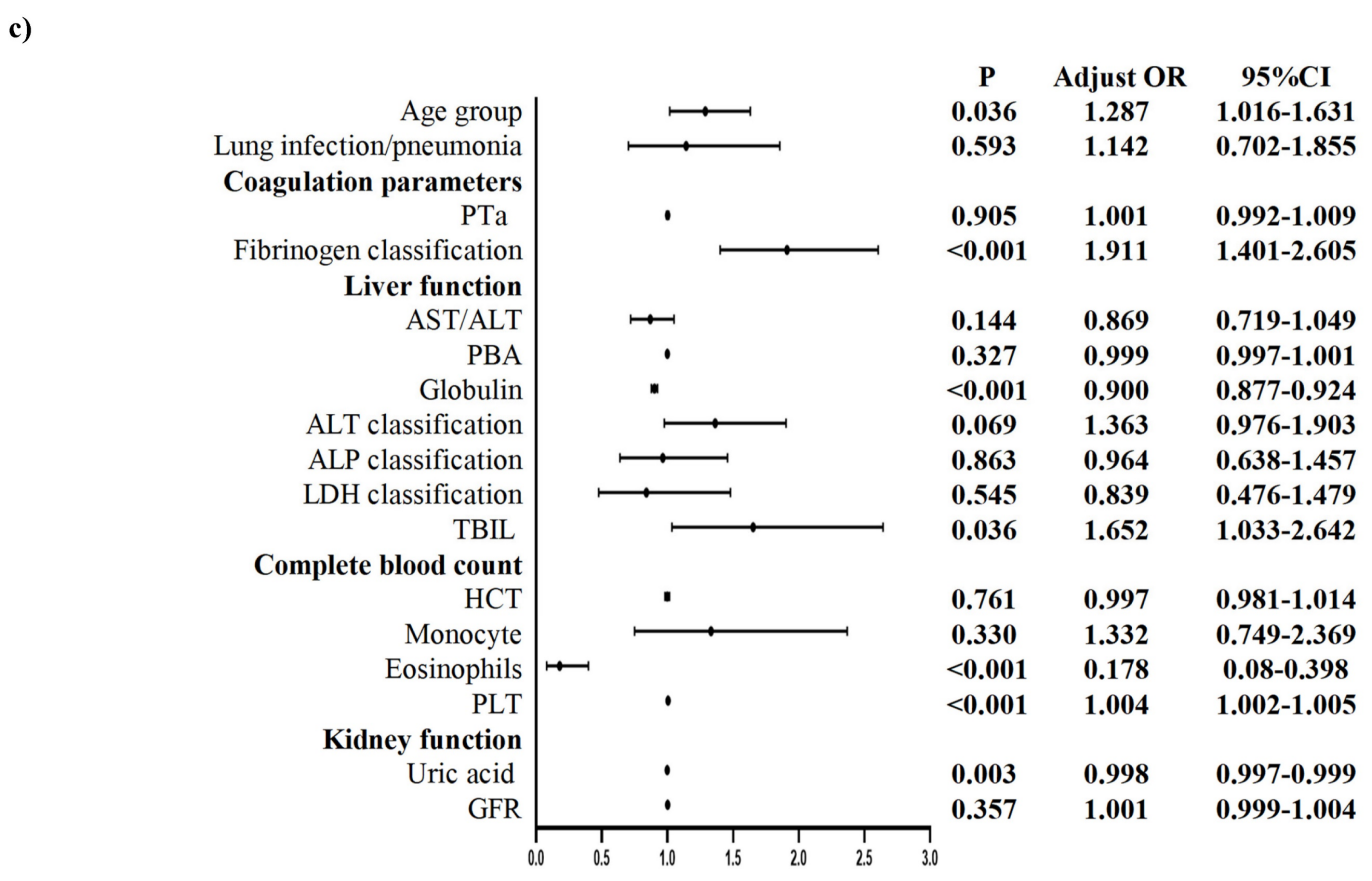

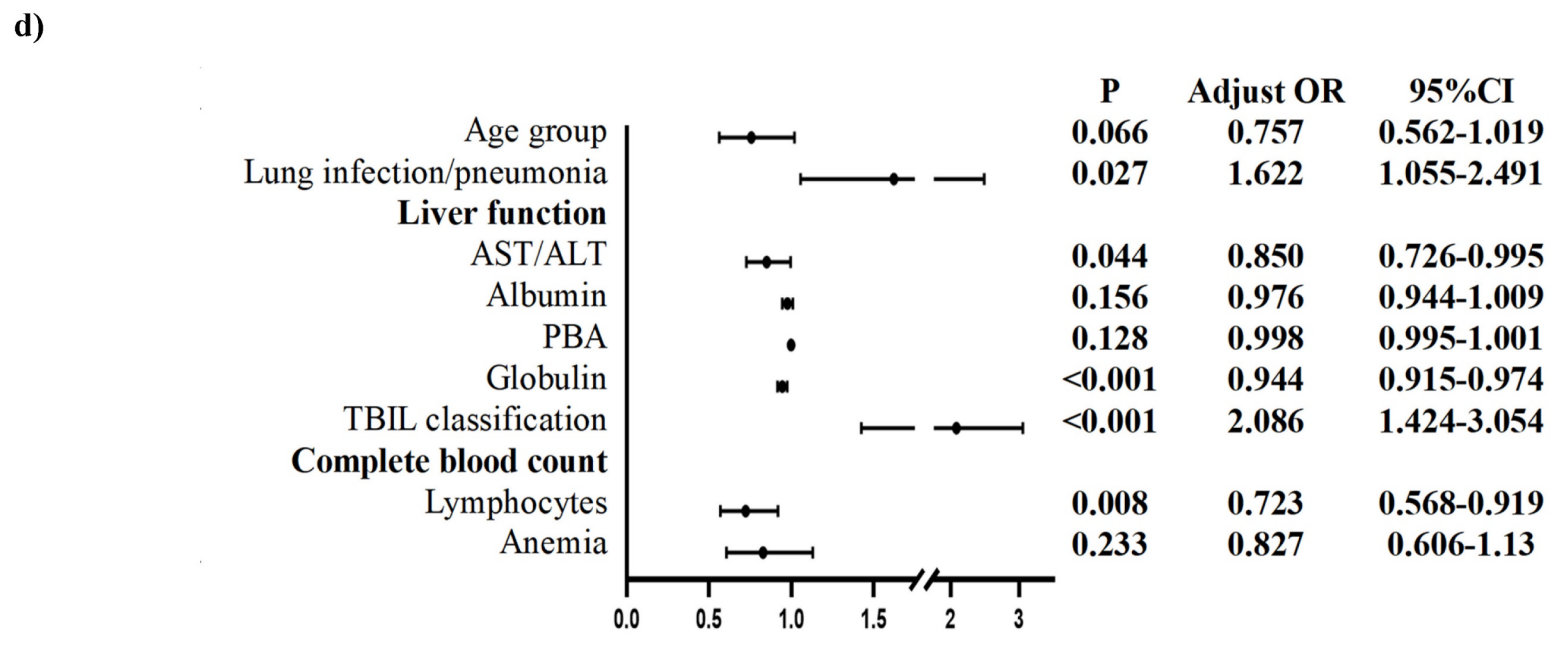
Subgroup analysis. **a** Preoperative multivariate logistic regression of cancer patients undergoing elective surgery in one month after infection with the Omicron variant and uninfected patients. ALT: Alanine aminotransferase; RBC: Red blood cell count; PLT: Platelet; PT: Thrombin time. *P* < 0.05 was statistically significant. **b** Postoperative multivariate logistic regression of cancer patients undergoing elective surgery in one month after infection with the Omicron variant and uninfected patients. ALP: Alkaline phosphatase; IBILI: Indirect bilirubin; PLT: Platelet; PT: Thrombin time; PTa: Prothrombin activity; RBC: Red blood cell count; PBA: Prealbumin; LDH: Lactate dehydrogenase; *P* < 0.05 was statistically significant. **c** Preoperative multivariate logistic regression of non-cancer patients undergoing elective surgery in one month after infection with the Omicron variant and uninfected patients. ALP: Alkaline phosphatase; ALT: Alanine aminotransferase; GFR: glomerular filtration rate; HCT: Hematocrit; PLT: Platelet; PTa: Prothrombin activity; RBC: Red blood cell count; PBA: Prealbumin; TBIL: Total bilirubin; LDH: Lactate dehydrogenase; *P* < 0.05 was statistically significant. **d** Postoperative multivariate logistic regression of non-cancer patients undergoing elective surgery in one month after infection with the Omicron variant and uninfected patients. ALT: Alanine aminotransferase; AST: Aspartate aminotransferase; PBA: Prealbumin; TBIL: Total bilirubin. *P* < 0.05 was statistically significant.

We further divided the patients into two subgroups for analysis: the tumor subgroup and the nontumor subgroup. According to the preoperative analysis of the tumor subgroup, compared with those in uninfected patients, the following findings were revealed in Group 2: globulin, monocyte, eosinophil, and platelet count (PLT) were considered significantly different (**Figure 5a**). Postoperative analysis of the tumor subgroup revealed that, compared with those in uninfected patients, the fibrinogen, PBA, and uric acid levels in Group 2 were significantly different (**Figure 5b**). In the perioperative period, the tumor subgroup had increased monocyte, PLT, and fibrinogen classification levels but exhibited a decrease in globulin, PBA, eosinophils, and uric acid. Notably, these changes were mild abnormalities. Compared with those of uninfected patients, the tumor subgroup showed no significant differences in preoperative coagulation or kidney function and minimal differences in the postoperative CBCs.

According to the preoperative analysis of the nontumor subgroup, compared with those in the uninfected patients, the fibrinogen level, globulin, TBIL, eosinophils, PLT, and uric acid levels were significantly different between the tumor patients and the uninfected patients (**Figure 5c**). Postoperative analysis of the nontumor subgroup revealed that the following factors were significantly different between patients in Group 2 and uninfected patients: lung infection/pneumonia, AST/ ALT, globulin, TBIL and lymphocyte count (**Figure 5d**). In the perioperative period, the nontumor subgroup showed an increase in fibrinogen level, lung infection/pneumonia status, TBIL, and PLT but a decrease in globulin and eosinophil levels. However, these changes were mild abnormalities.

## Discussion

In the early stages of the COVID-19 pandemic, the dominant SARS-CoV-2 variants exhibited increased pathogenicity. Therefore, El-Boghdadly et al. recommended a 7-week delay post infection before surgery to allow the perioperative risk to return to baseline 15. However, the Omicron (B.1.1.529) variant and its subvariants seem to cause less serious illness than earlier types of coronavirus 16. A retrospective study with a limited sample size conducted by Sridhar et al. suggested that patients who have recovered from asymptomatic and mild COVID-19 infections can safely undergo elective general surgical procedures after a minimum waiting period of two weeks 17. Barie et al. emphasized the significance of evaluating the feasibility of reducing the waiting period for elective surgeries in asymptomatic or vaccinated patients, given that the Omicron variant/subvariant usually results in less severe infections with fewer organ-related complications 18. In our study, the patients were predominantly infected with the Omicron-BA.5.2 variant, with some cases involving Omicron-BF7. We scheduled the elective surgery based on a collaborative decision involving the patient, surgeon, and anesthesiologist 19. Most of the elective surgeries were performed four weeks after infection with the Omicron variant.

To the best of our knowledge, our study is the first to show, from the perspective of coagulation parameters, liver function, CBC, and kidney function, that Omicron variant infections have a minimal impact on various parameters in patients undergoing elective surgery one month after the Omicron variant infection, regardless of whether the operation was preoperative or in the context of surgical trauma. We also analyzed patient subgroups by dividing the patients into tumor and nontumor subgroups, yielding similar conclusions. Our findings suggest that, in comparison to those of uninfected patients, the main effects of the Omicron variant on patients during the perioperative period, one month post-infection, are as follows: a mild increase in blood coagulability, primarily characterized by a slight increase in fibrinogen levels; a mild decrease in albumin, globulin, and AST/ALT; slight elevation in ALT levels; a mild decrease in WBC and eosinophil counts; mild anemia in the CBC; and a mild decrease in uric acid in kidney function. Remarkably, no statistically significant differences were found in either the mortality rate or LOS between the two groups. Subgroup analyses showed that, compared with that in uninfected patients, the Omicron variant did not significantly affect kidney function in the tumor group during the perioperative period, while the effects on coagulation, liver function, and CBC were minor. Similar hematological trends were also detected in the nontumor subgroup. We compared the incidences of lung infection/pneumonia, pleural effusion, DVT, and anemia as postoperative outcomes. Only lung infection/pneumonia had a significant association with an OR of 1.508, while the remaining indicators had *p* values above 0.05, indicating no significant associations.

### Impact of Omicron variant on coagulation parameters in patients undergoing elective surgery one month after infection with the Omicron variant

Zhang J et al. reported a significant occurrence of coagulopathy in patients with Omicron variant infection 12. For instance, even though the D-D levels of most patients are within the normal range, abnormally high values were detected in 11.8% of the patients. Similar coagulation events include prolonged PT and APTT 12. Similarly, Gupta et al. reported that COVID-19 is associated with markedly elevated D-D and fibrinogen levels, prolonged PT, and APTT in patients at risk of developing arterial and venous thrombosis 20. This finding is consistent with our findings. Our study demonstrated that the impact of Omicron variant infection on coagulation in patients one month after infection was characterized by slight increases in fibrinogen and PT compared to those in uninfected patients, both before and after surgery. In addition, the subgroup analysis revealed a twofold increase in fibrinogen classification in both the tumor and nontumor subgroups. In contrast, the other coagulation indices did not significantly differ. Notably, the Omicron variant had little impact on the pre- or postoperative coagulation parameters in patients who underwent elective surgery one month after Omicron variant infection.

Ferrandis R et al. reported that hypercoagulability may persist for one to three months post infection and even up to six months 10. A study by Morales-Garc et al. suggested that patients are still in a mild inflammatory state up until six to eight weeks after recovering from COVID-19 21. Given that most of our patients were infected with the Omicron variant one month earlier, it is likely that they were still in a mild inflammatory state. In COVID-19 patients, hypercoagulopathy can occur through infection-induced dysfunctions that develop in endothelial cells during disease pathogenesis, overproduction of thrombin, blockade of fibrinolysis, or increased viscosity due to hypoxia 22.

### Impact of the Omicron variant on liver function in patients undergoing elective surgery one month after infection

A meta-analysis by Kumar-M et al. showed that derangement of liver functions was common in COVID-19 patients. The most frequent abnormalities included hypoalbuminemia, followed by elevated GGT, ALT, bilirubin, and ALP levels 23. According to a retrospective study of the Omicron variant by Deng et al., liver function abnormalities were predominantly characterized by mild elevations in markers of choanocytes or biliary duct injury (TBIL, ALP, or GGT) in Omicron-infected patients 13. Another retrospective study by Feng et al. revealed a greater risk of liver injury in Omicron-infected patients, with liver injury observed in up to 23.7% of COVID-19 patients. The liver function tests in COVID-19 patients with liver injury revealed mildly elevated serum AST and ALT levels. GGT, ALP, TBIL, IBILI, and DBIL were also found to be higher in patients with liver injury 24. The prognosis of patients with COVID-19 complicated with liver injury was generally good, with 95.6% of patients having liver function tests that returned to normal two months after discharge 24. Our findings indicate that, compared with that in uninfected patients, the impact of the Omicron variant on liver function in patients one month after infection is primarily characterized by mild decreases in the serum ALB concentration, globulin, and PBA and slight increases in the serum ALT concentration, ALP, LDH, and AST both before and after surgery. Although full recovery of liver function may not be possible within this time frame, the differences are minimal.

The pathogenic mechanism of liver involvement caused by SARS-CoV-2 may be multifactorial and include viral liver infection, systemic inflammation caused by cytokine storms, drug-induced liver injury, and hypoxemia associated with pneumonia 25. Their study indicated that the recovery of liver function may require at least 14 days, consistent with the findings of a follow-up study conducted by An YW et al. 26. Deng et al. identified inflammation as an independent factor associated with liver injury 13. Our patients' inability to fully recover their liver function to baseline levels may be because they were in a mild inflammatory state one month after Omicron variant infection.

### Impact of the Omicron variant on CBC in patients undergoing elective surgery one month after infection with the Omicron variant

Previous studies have reported that leukocytosis, lymphocytosis, or decreased hemoglobin and eosinophils are present after recovery from COVID-19 27. A prospective cohort study on the Omicron variant by Teng L et al. suggested that hemoglobin, WBC, PLT, and C reactive protein (CRP) did not significantly differ six months after Omicron infection 28. Our study revealed that, compared to those of uninfected patients, after one month of Omicron variant infection and before surgery, the WBC, CRP, and neutrophil counts recovered to baseline levels; additionally, the hemoglobin, RBC, lymphocyte, and eosinophil counts were lower, and the PLT and monocyte counts were slightly greater. Overall, the impact of the Omicron variant on CBC parameters in patients undergoing elective surgery one month after infection is minimal, regardless of their tumor status or surgical experience.

Wang et al. reported a reduced hemoglobin concentration (< 110 g/L) in 19% of hospitalized patients 29. Among the patients in this study, the incidence rate of preoperative anemia in infected patients one month after Omicron infection was 34.18%, whereas in the uninfected group, it was 27.23%. Lechner-Scott J et al. proposed that circulating inflammatory markers are still present in post-COVID-19 patients and that the long COVID syndrome duration is associated with residual inflammation 30. Patients remain in a mild inflammatory state one month after Omicron variant infection, which directly or indirectly suppresses RBC production and shortens the lifespan of RBCs 31, thus leading to inflammatory anemia. However, because the inflammation is mild, the anemia is also slight. The exact mechanism underlying the eosinopenia may involve eosinophils migrating to the inflammatory site, inhibiting eosinophil release from the bone marrow, and suppressing eosinophilopoiesis 27. The slight increase in the PLT after one month of Omicron variant infection in our patients is attributed to cytokine storm-induced stimulation of megakaryocytes 33.

### Impact of the Omicron variant mutations on kidney function in patients who underwent elective surgery one month after infection

A prospective cohort study by Teng et al. on the Omicron variant revealed no statistically significant differences in BUN, UA, or other indicators during a 6-month follow-up period 28. This study revealed a downward trend in kidney function after Omicron variant infection, and the kidney function returned to the baseline level within six months. Moreover, a mild inflammatory state was indicated in patients one month after Omicron variant infection 28. In the present study, we found that one month after Omicron variant infection, compared to uninfected patients, the infected patients only had a slight decrease in uric acid levels, whether preoperative or postoperative or in subgroup analysis. Our study suggested that the impact of the Omicron variant on kidney function is insignificant in patients who underwent elective surgery one month after infection, regardless of tumor status or experience of surgical trauma.

These changes could be attributed to two factors. First, COVID-19 vaccination may have protected patients infected with the Omicron variant 34,35. Le ST et al. reported that previous SARS-CoV-2 vaccination may have a protective effect against perioperative complications 36. Additionally, McMahan et al. reported that Omicron causes reduced inflammatory processes and attenuated replication in mice and hamsters 37. A study by Morales-Garc et al. indicated that patients remain in a state of mild inflammation six to eight weeks after recovering from COVID-19 21. Most of the patients in this study were in a post recovery phase of approximately four weeks and still exhibited a mild inflammatory state.

In this retrospective analysis, we collected the medical records and laboratory data of patients who underwent elective surgery, one month after confirmation of Omicron variant infection or without infection. Nevertheless, it is essential to acknowledge the limitations of this study. As a retrospective study, some data may be incomplete, including the data of 190 patients whose preoperative laboratory test records were unavailable (these tests were conducted outside our hospital). Additionally, given the single-center and retrospective nature of this case‒control study, potential confounding factors may exist. Furthermore, we did not perform whole-genome sequencing of the coronavirus for each patient in our hospital, and it is impossible to definitively ascertain the specific subtypes of infection for each patient.

## Conclusion

Compared with uninfected patients, patients who underwent elective surgery one month after Omicron variant infection exhibited minimal changes in perioperative coagulation parameters, liver function, CBC counts, and kidney function. Additionally, no significant differences in postoperative mortality or LOS were observed between the two groups.

## Author contributions

Contributed to the conception and design of the study: Qingxiang Mao, Rui Li, and Haoyu Pei.

Contributed to the acquisition and analysis of data: Rui Li and Haoyu Pei.

Responsible for statistical analysis: Rui Li and Xiaojuan Xiong.

Oversaw project conception and acts as overall guarantor: Qingxiang Mao.

Wrote and revised the manuscript: Xiaojuan Xiong. Read and approved the final manuscript, offering critical feedback to all members of the authorship group.

## Figures and Tables

**Table 1:** Summary of patient characteristics.

Influencing factor	Reference Range	Preoperation				Postoperation		
		Group 1(3097)		Group 2(1932)	*P*	Group 1(3097)	Group 2(1932)	*P*
Age (year)		49.18±0.3		46.82±0.41	< 0.001	49.18±0.3	46.82±0.41	< 0.001
**Coagulation parameters**								
D-D (μg/L)	0-232	258.41±10.32		356.05±28.91	< 0.001	1541.17±380.58	1707.54±257.95	0.766
APTT(s)	24-40	30.7±0.06		30.2±0.09	< 0.001	29.29±0.11	29.28±0.16	< 0.001
TT (s)	11-18	14.03±0.07		14.12±0.14	0.535	13.47±0.09	13.88±0.36	0.204
PTa (%)	70-120	104.72±0.27		103.66±0.37	0.022	91.37±0.4	90.2±0.61	0.998
PT (s)	9.4-13.8	11.1±0.02		11.29±0.03	< 0.001	12.18±0.04	12.41±0.06	< 0.001
Fibrinogen (g/L)	2-4	3.12±0.02		3.27±0.02	< 0.001	3.53±0.03	3.66±0.04	0.059
**Liver function**								
AST/ALT	0.5-2	1.33±0.01		1.25±0.01	< 0.001	1.59±0.02	1.49±0.03	0.007
GGT (U/L)	7-45		37.43±2.1	35.31±1.35	0.445	38.41±2.13	39.35±2.41	0.786
Albumin/Globulin	1.2-2.4		1.36±0	1.42±0.01	< 0.001	1.3±0.01	1.31±0.01	0.532
Albumin (g/L)	40-55		39.73±0.08	39.61±0.11	0.375	34.17±0.13	33.33±0.18	< 0.001
ALT (U/L)	7-40		23.77±0.68	28.17±1.55	0.009	29.13±1.55	37.44±5.6	0.153
IBILI (μmol/L)	0-16.2		11.01±0.14	10.44±0.22	0.518	15.2±0.49	13.65±0.52	0.047
ALP (U/L)	50-135		95.73±1.15	103.59±1.51	< 0.001	91.91±2.14	93.49±1.96	0.633
DBIL (μmol/L)	0-6.8		2.49±0.12	2.66±0.22	0.5	3.82±0.2	4.92±0.57	0.068
PBA (mg/L)	160-384		243.81±1.21	237.29±1.51	< 0.001	200.76±1.76	191.1±2.62	0.002
Globulin (g/L)	20-40		29.61±0.08	28.39±0.1	< 0.001	27.03±0.12	26.45±0.16	0.004
LDH (U/L)	120-250		170.58±1.21	179.82±2.65	< 0.001	188.65±4.72	199.04±8.56	0.249
AST (U/L)	13-35		24.86±0.39	28.98±2.2	0.026	37.23±3.1	44.92±6.71	0.298
TBIL (μmol/L)	1.7-2.3		13.77±0.25	13.34±0.43	0.35	19.55±0.6	19.72±1.1	0.881
TP (g/L)	65-85		69.33±0.12	68.02±0.16	< 0.001	61.5±0.21	60.35±0.29	< 0.001
**Complete Blood count**								
Hemoglobin (g/L)	115-150		130.87±0.34	129.32±0.4	0.003	120±0.42	118.11±0.58	0.008
RBC (*10^12/L)	3.8-5.1		4.38±0.01	4.34±0.01	0.01	4±0.01	3.94±0.02	0.004
MCH (pg)	27-34		29.96±0.05	29.94±0.06	0.852	30.08±0.06	30.15±0.08	0.48
MCHC (g/L)	316-351		326.58±0.22	327.45±0.25	0.009	326.91±0.25	327.62±0.35	0.098
HCT (%)	35-45		40.01±0.09	39.45±0.11	< 0.001	36.66±0.12	36.01±0.16	0.205
RDW (fL)	11.6-15.3		28.54±0.3	28.57±0.37	0.612	29.13±0.36	28.36±0.49	0.204
MCV (fL)	82-100		91.65±0.12	91.38±0.16	0.176	91.95±0.15	91.95±0.2	0.998
PLT (*10^12/L)	125-350		221.82±1.36	232.55±1.79	< 0.001	203.37±1.57	206.61±2.39	0.24
Large platelet ratio (%)	13-43		232.55±1.79	29.91±0.22	0.021	30.85±0.2	30.96±0.28	0.765
PDW (fL)	9-17		13.91±0.05	13.96±0.07	0.612	13.45±0.06	13.5±0.08	0.642
MPV (fL)	9.4-18.3		10.68±0.02	10.58±0.03	0.014	10.73±0.03	10.75±0.04	0.65
WBC (*10^12/L)	3.5-9.5		6.44±0.05	6.46±0.06	0.722	10.19±0.08	9.88±0.11	0.029
Monocyte (*10^12/L)	0.1-0.6		0.45±0	0.47±0	0.003	0.632±0.006	0.631±0.009	0.969
Lymphocytes (*10^12/L)	1.1-3.2		1.71±0.01	1.68±0.02	0.245	1.2±0.01	1.12±0.02	< 0.001
Neutrophils (*10^12/L)	1.8-6.3		4.09±0.04	4.16±0.05	0.276	8.26±0.08	8.04±0.11	0.11
Basophils (*10^12/L)	0-0.06		0.03	0.03	< 0.001	0.024	0.02	< 0.001
Eosinophils (*10^12/L)	0.02-0.52		0.14	0.12	< 0.001	0.069±0.002	0.061±0.003	0.022
CRP (mg/L)	0-8		4.62±0.36	5.21±0.43	0.295	28.59±0.88	31.49±1.31	0.067
**Renal function**								
GFR (ml/min/1.73m2)			144.79±0.8	153.16±1.12	< 0.001	152.14±1.45	155.88±2.19	0.142
Creatinine (μmol/L)	41-81		62.85±0.91	60.3±1.18	0.083	64.2±1.5	67.19±3.64	0.374
Urea (mmol/L)	3.1-8.8		5.15±0.04	5.04±0.06	0.069	4.86±0.07	4.97±0.15	0.49
Urea/Creatinine	5-50		21.71±0.15	22.09±0.2	0.134	20.22±0.23	20.23±0.31	0.98
Uric acid (μmol/L)	155-357		329.37±1.8	308.03±2.18	< 0.001	278.79±2.73	262.86±3.9	< 0.001
								

Group 1: not infected with the Omicron variant; Group 2: one month after Omicron variant infection). ALP: Alkaline phosphatase; ALT: Alanine aminotransferase; APTT: Activated partial thromboplastin time; AST: Aspartate aminotransferase; CHD: Coronary heart disease; COPD: Chronic obstructive pulmonary disease; CRP: C reactive protein; DBIL: Direct bilirubin; DVT: Deep vein thrombosis; GFR: glomerular filtration rate; GGT: gamma‐glutamyl transpeptidase; HCT: Hematocrit; IBILI: Indirect bilirubin; MCH: mean corpuscular hemoglobin; MCHC: mean corpuscular hemoglobin concentration; MCV: mean corpuscular volume; MPV: mean platelet volume; PDW: platelet distribution width; P-LCR: platelet larger cell ratio; PLT: Platelet; PT: Thrombin time; PTa: Prothrombin activity; RBC: Red blood cell count; TBIL: Total bilirubin; TT: Thrombin time; UA: Uric acid; WBC: White blood cell count; PBA: Prealbumin; LDH: Lactate dehydrogenase; TP: Total protein; RDW: Red cell distribution width. *P* < 0.05 was statistically significant.

**Table 2:** Preoperative and postoperative univariate analysis of patients undergoing elective surgery one month after infection with the Omicron variant.

Influencing factor	Pre-operation		Post-operation	
	*X^2^*	*P*	*X^2^*	*P*
Hypertension	0.1	0.75	0.1	0.75
Diabetes	0.6	0.43	0.6	0.43
CHD	0.88	0.35	0.88	0.35
COPD	0.23	0.63	0.23	0.63
Lung infection/pneumonia	6.81	0.009	6.81	0.009
Pleural effusion	0.61	0.44	0.61	0.44
Gender	0.04	0.84	0.04	0.84
Age grading (≥60 years)	7.93	0.005	7.93	0.005
DVT	2.08	0.15	0.17	0.68
D-D group (>400 ug/L)	13.22	< 0.001	7.9	0.005
APTT classification (>40s)	35.38	< 0.001	0.14	0.69
TT classification (>18s)	0.27	< 0.001	0.44	0.51
PTa classification (>120%)	0.01	0.92	2.81	0.09
PT classification (>13.8s)	2.16	0.14	8.27	0.004
Fibrinogen classification (>4g/L)	21.85	< 0.001	8.88	0.003
GGT classification	0.4	0.53	2.29	0.13
Albumin classification	0.02	0.9	0.96	0.33
ALT classification	13.78	< 0.001	0.76	0.38
ALP classification	10.86	0.001	2.45	0.12
LDH classification	9.19	0.002	3.09	0.08
AST classification	2.47	0.12	1.4	0.24
TBIL classification	2.61	0.11	11.72	0.001
Renal insufficiency	0.005	0.94	0.28	0.6
Anemia	8.42	0.004	11.91	< 0.001
PLT classification (>100*10^12^/L)	< 0.001	0.99	2.43	0.12

ALP: Alkaline phosphatase; ALT: Alanine aminotransferase; APTT: Activated partial thromboplastin time; AST: Aspartate aminotransferase; CHD: Coronary heart disease; COPD: Chronic obstructive pulmonary disease; DVT: Deep vein thrombosis; GGT: gamma‐glutamyl transpeptidase; PLT: Platelet; PT: Thrombin time; PTa: Prothrombin activity; TBIL: Total bilirubin; TT: Thrombin time; LDH: Lactate dehydrogenase; TP: Total protein.* P* < 0.05 was statistically significant.

**Table 3:** The incidence of complications after elective surgery.

Influencing factor	Post operation	
	Group 1	Group 2
Mortality	4(0.13%)	7(0.36%)
Lung infection/pneumonia	105(3.39%)	94(4.87%)
Pleural effusion	24(0.77%)	19(0.98%)
DVT	36(1.16%)	25(1.29)
Renal insufficiency	113(8.2%)	56(7.58%)
Anemia	1122(54.38%)	675(61.26%)

Group 1: not infected with the Omicron variant; Group 2: one month after Omicron variant infection. DVT: Deep vein thrombosis.

## Abbreviations

ALP: Alkaline phosphatase
ALT: Alanine aminotransferase
APTT: Activated partial thromboplastin time
AST: Aspartate aminotransferase
CBC: complete blood count
CHD: Coronary heart disease
CI: confidence interval
COPD: Chronic obstructive pulmonary disease
CRP: C reactive protein
DBIL: Direct bilirubin
DVT: Deep vein thrombosis
GFR: glomerular filtration rate
GGT: gamma‐glutamyl transpeptidase
HCT: Hematocrit
Hb: hemoglobin
IBILI: Indirect bilirubin
LOS: length of stay
MCH: mean corpuscular hemoglobin
MCHC: mean corpuscular hemoglobin concentration
MCV: mean corpuscular volume
MPV: mean platelet volume
PBA: Prealbumin
PDW: platelet distribution width
P-LCR: platelet larger cell ratio
PLT: platelet
PT: prothrombin time
PTa: Prothrombin activity
RBC: Red blood cell count
TBIL: Total bilirubin
TT: Thrombin time
UA: Uric acid
WBC: White blood cell count
LDH: Lactate dehydrogenase
TP: Total protein
RDW: Red cell distribution width

## References

[1] Harenberg J, Favaloro E (2020). COVID-19: progression of disease and intravascular coagulation - present status and future perspectives. Clin Chem Lab Med.

[4] World Health Organization 2021. Classification of Omicron (B.1.1.529): SARS-CoV-2 variant of concern. https://www.who.int/news/item/26-11-2021classification-of-omicron-(b.1.1.529)-sars-cov-2-variant-of-concern.

[9] Madhi SA, Kwatra G, Myers JE (2022). Population immunity and COVID-19 severity with Omicron variant in South Africa. N Engl J Med.

[10] Ferrandis R, Llau JV, Afshari A, Douketis JD, Gómez-Luque A, Samama CM (2021). Management of perioperative thromboprophylaxis for surgery following COVID-19: an expert-panel survey. Br J Anaesth.

[11] Wolff D, Nee S, Hickey NS, Marschollek M (2021). Risk factors for COVID-19 severity and fatality: a structured literature review. Infection.

[12] Zhang J, Chen N, Zhao D, Zhang J, Hu Z, Tao Z (2022). Clinical characteristics of COVID-19 patients infected by the Omicron variant of SARS-CoV-2. Front Med (Lausanne).

[13] Deng H, Mai Y, Liu H, Guan J (2023). Clinical characteristics of liver injury in SARS-CoV-2 Omicron variant- and Omicron subvariant-infected patients. Ann Hepatol.

[14] Hemoglobin concentrations for the diagnosis of anaemia and assessment of severity (2011). Vitamin and Mineral Nutrition Information System. Geneva, World Health Organization.

[15] El-Boghdadly K, Cook TM, Goodacre T (2021). SARS-CoV-2 infection, COVID-19 and timing of elective surgery: A multidisciplinary consensus statement on behalf of the Association of Anaesthetists, the Centre for Peri-operative Care, the Federation of Surgical Specialty Associations, the Royal College of Anaesthetists and the Royal College of Surgeons of England. Anaesthesia.

[16] Maslo C, Friedland R, Toubkin M (2022). Characteristics and Outcomes of Hospitalized Patients in South Africa During the COVID-19 Omicron Wave Compared With Previous Waves. JAMA.

[17] Sridhar RP, Titus DK, Surendran S (2022). Postoperative outcomes in patients undergoing elective general surgery after recovery from Covid-19 at a tertiary care centre: A one-year case series. Natl Med J India.

[19] Barnes GD, Burnett A, Allen A (2022). Thromboembolic prevention and anticoagulant therapy during the COVID-19 pandemic: updated clinical guidance from the anticoagulation forum. J Thromb Thrombolysis.

[20] Gupta A, Madhavan MV, Sehgal K (2020). Extrapulmonary manifestations of COVID-19. Nat Med.

[21] Morales-García D, Docobo-Durantez F, Capitán Vallvey JM (2022). Consensus of the ambulatory surgery commite section of the Spanish Association of Surgeons on the role of ambulatory surgery in the SARS-CoV-2 pandemic. Cir Esp.

[22] Mustafa U Ğ. U. Z, and Burak EŞKUT. “Covid 19 enfeksiyon tedavisi.” Medical Research Reports 3.Özel Sayı (2020): 17-31.

[23] Kumar-M P, Mishra S, Jha DK (2020). Coronavirus disease (COVID-19) and the liver: a comprehensive systematic review and meta-analysis. Hepatol Int.

[24] Feng Y, Liu Y, Zhao Q (2023). Liver injury in patients with COVID-19: A retrospective study. Int J Med Sci.

[25] Yu D, Du Q, Yan S (2021). Liver injury in COVID-19: clinical features and treatment management. Virol J.

[26] An YW, Song S, Li WX (2021). Liver function recovery of COVID-19 patients after discharge, a follow-up study. Int J Med Sci.

[27] Pereira-Roche N, Roblejo-Balbuena H, Marín-Padrón LC (2022). Hematological alterations in patients recovered from SARS-CoV-2 infection in Havana, Cuba. MEDICC Rev.

[28] Teng L, Song X, Zhang M (2023). The pattern of cytokines expression and dynamic changes of renal function at 6 months in patients with Omicron COVID-19. J Med Virol.

[29] Huang Y, Tu M, Wang S (2020). Clinical characteristics of laboratory confirmed positive cases of SARS-CoV-2 infection in Wuhan, China: A retrospective single center analysis. Travel Med Infect Dis.

[30] Lechner-Scott J, Levy M, Hawkes C, Yeh A, Giovannoni G (2021). Long COVID or post COVID-19 syndrome. Mult Scler Relat Disord.

[31] Branchford BR, Carpenter SL (2018). The role of inflammation in venous thromboembolism. Front Pediatr.

[32] Butterfield JH (2007). Treatment of hypereosinophilic syndromes with prednisone, hydroxyurea, and interferon. Immunol Allergy Clin North Am.

[33] Amgalan A, Othman M (2020). Hemostatic laboratory derangements in COVID-19 with a focus on platelet count. Platelets.

[34] Cortellini A, Tabernero J, Mukherjee U (2023). SARS-CoV-2 omicron (B.1.1.529)-related COVID-19 sequelae in vaccinated and unvaccinated patients with cancer: results from the OnCovid registry. Lancet Oncol.

[35] COVIDSurg Collaborative, GlobalSurg Collaborative (2021). SARS-CoV-2 vaccination modelling for safe surgery to save lives: data from an international prospective cohort study. Br J Surg.

[36] Le ST, Kipnis P, Cohn B, Liu VX (2022). COVID-19 vaccination and the timing of surgery following COVID-19 infection. Ann Surg.

[37] McMahan K, Giffin V, Tostanoski LH (2022). Reduced pathogenicity of the SARS-CoV-2 omicron variant in hamsters. Med.

